# Retraction: Spermatogonial stem-cell-derived neural-like cell transplantation enhances the functional recovery of a rat spinal cord injury model: characterization of evoked potentials

**DOI:** 10.3389/fnins.2024.1442958

**Published:** 2024-06-11

**Authors:** Frontiers Editorial Office

The journal retracts the 2023 article cited above.

This article is retracted upon request from the first author Xinyu Guo. Following publication, one of the corresponding authors, Dingjun Hao contacted the publisher and stated that he did not participate in the research and the writing of this article. He was listed as the author without his approval and knowledge. An investigation conducted in accordance with Frontiers' policies found that parts of the raw data associated with the study are not available. Given the concerns regarding authorship, and the lack of raw data, the editors no longer have confidence in the findings presented in the article and the article is therefore retracted. The first author Xinyu Guo apologizes to Prof. Dingjun Hao and the publisher for the inconvenience caused.

The authors have agreed to this retraction.

This retraction was approved by the Chief Editors of Frontiers in Neuroscience and the Chief Executive Editor of Frontiers.

